# Advances in Migratory Plant Endoparasitic Nematode Effectors

**DOI:** 10.3390/ijms25126435

**Published:** 2024-06-11

**Authors:** Yang Lu, Sihua Yang, Wenhao Chen, Hui Xie, Chunling Xu

**Affiliations:** Research Center of Nematodes of Plant Quarantine, Guangdong Province Key Laboratory of Microbial Signals and Disease Control, Department of Plant Pathology, College of Plant Protection, South China Agricultural University, Guangzhou 510642, China

**Keywords:** plant-parasitic nematodes, migratory endoparasitism, effectors

## Abstract

Unlike sedentary plant-parasitic nematodes, migratory plant endoparasitic nematodes (MPENs) are unable to establish permanent feeding sites, and all developmental stages (except eggs) can invade and feed on plant tissues and can be easily overlooked because of the unspecific symptoms. They cause numerous economic losses in agriculture, forestry, and horticulture. In order to understand the pathogenetic mechanism of MPENs, here we describe research on functions and host targets focused on currently identified effectors from six MPENs, namely *Radopholus similis*, *Pratylenchus* spp., *Ditylenchus destructor*, *Bursaphelenchus xylophilus*, *Aphelenchoides besseyi*, and *Hirschmanniella oryzae*. This information will provide valuable insights into understanding MPEN effectors and for future fostering advancements in plant protection.

## 1. Introduction

Plant-parasitic nematodes are one of the pathogens that cause plant infectious diseases, with estimated global economic losses reaching as high as $173 billion annually [1]. The parasitic nematodes can be classified into ectoparasitic, semi-endoparasitic, and endoparasitic nematodes according to the mode of parasitism, of which the endoparasitic nematodes can be further divided into migratory and sedentary endoparasites [2]. Migratory plant endoparasitic nematodes (MPENs) are incapable of inducing the formation of feeding sites within host plants, primarily changing their feeding locations by migrating between plant cells, with all life stages observable within host tissues [3]. Among the top ten plant-parasitic nematodes known to cause serious crop diseases, 50% of them are migratory parasitic nematodes encompassing *Radopholus similis*, *Pratylenchus* spp., *Ditylenchus* spp., *Bursaphelenchus xylophilus*, and *Aphelenchoides besseyi* [4].

The burrowing nematode, *R. similis*, is a significant quarantine pest worldwide, notorious for infesting the phloem and cortex of plant roots. It displays a wide range of host species susceptibility, affecting over 360 plant species, including economically important crops such as bananas, citrus fruits, and various ornamental plants. The economic impact of its infestation on bananas alone can be substantial, reaching up to $187 million [5,6,7]. *Pratylenchus* spp., also known as root lesion nematodes, ranks as the second most formidable nematode pest of cultivated crops globally. All species within this genus have the potential to cause damage to host plants by feeding and moving within the root cortex, posing a significant threat to global agricultural economies [8,9,10]. *Ditylenchus destructor*, a species within the *Ditylenchus* genus, is listed as an important quarantine organism globally, severely impacting the production of sweet potatoes and potatoes, causing yield losses ranging from 20% to 50% [11,12]. *B. xylophilus* can induce devastating diseases in pine trees, often leading to the death of infected trees and resulting in irreparable economic losses annually. It has become one of the most serious coniferous diseases worldwide, affecting pine species extensively [13,14]. *A. besseyi* is a prominent nematode pest in rice cultivation, capable of causing yield reductions ranging from 10% to 70% under severe infestation. Moreover, it has been implicated in soybean green stem and foliar retention syndrome, causing yield losses of up to 60% in soybean cultivation regions [15,16,17,18]. Additionally, *Hirschmanniella oryzae* is a significant migratory endoparasitic nematode, contributing to rice yield losses [19,20]. The management of plant-parasitic nematodes primarily relies on chemical interventions. However, due to the adverse impacts of certain nematocides on human health, animal welfare, and the environment, the production and use of specific nematocides have been banned [21,22]. Consequently, the emergence of RNA interference (RNAi) technology, with its target specificity and ecological compatibility, has introduced a novel approach to combat plant-parasitic nematodes [23,24,25]. RNAi shows promising applications in controlling sedentary plant-parasitic nematodes and has yielded favorable outcomes in combating MPENs such as *R. similis*, *A. besseyi*, and *B. xylophilus* [26,27,28,29,30,31,32,33,34].

Plant-parasitic nematodes inflict damage on plants not only through mechanical means and nutrient absorption but also through the chemical potency of their secretions. Nematode effectors, including proteins and various small biological molecules, are excreted by esophageal glands, head sensilla, tail sensilla, and the body wall, among other structures. These effectors can alter the structure and function of host cells, playing a crucial role in the nematode’s interaction with host plants. Typically, they possess signal peptides without transmembrane domains and can penetrate various parts of plant cells [35,36]. Effector molecules of plant-parasitic nematodes have garnered significant attention from scholars worldwide in recent years. While substantial research has focused on elucidating the effectors of sedentary plant-parasitic nematodes, studies on the effectors of migratory plant endoparasitic nematodes remain relatively scarce [37,38,39,40,41]. Although efforts have been made to identify the effectors of migratory plant endoparasitic nematodes through cloning endeavors, they have not received as much attention as their sedentary counterparts.

In this review, we primarily concentrate on six MPENs: *R. similis*, *Pratylenchus* spp., *D. destructor*, *B. xylophilus*, *A. besseyi*, and *H. oryzae*. We discuss the synthesis, secretion, functions, and interactions with host plants of effectors derived from migratory plant endoparasitic nematodes. This elucidation aims to foster a deeper understanding of these effectors and provide a basis for identifying potential targets for their control.

## 2. Synthesis and Secretion of Effectors by Migratory Plant Endoparasitic Nematodes

### 2.1. The Synthetic Sites of Effectors in Migratory Plant Endoparasitic Nematodes

Effectors originating from the esophageal glands of plant-parasitic nematodes are primarily associated with pathogenicity, exerting control over the host’s immune responses and assuming pivotal roles in parasitic interactions, whereas effectors derived from alternate sites may incite physiological reactions within plants [42]. Some MPEN effectors display specific localization within the esophageal glands, such as the five pectate lyase genes (*Rs-Pel-1*, *Rs-Pel-2*, *Rs-Pel-3*, *Rs-Pel-4*, *Rs-Pel-5*) of *R. similis* [43], the serine carboxypeptidase gene AbSCP1 of *A. besseyi* [44], the cellulase gene *Bx-eng-1* of *B. xylophilus* [45], the venom allergen-like gene *DdVAP2* of *D. destructor* [46], and the pectin methylesterase gene *Pp-pme* of *P. penetrans* [47], among others. However, besides the esophageal glands, effectors are also secreted in other parts of the nematode’s body. For example, the effector BxSCD1 of *B. xylophilus* is not only localized in the esophageal glands but also detected in the intestine with hybridization signals [48]. Additionally, the cellulase gene *Pv-eng-5* of *P. vulnus* exhibits hybridization signals in the intestine [49]. Ding et al. demonstrated the immunolocalization of the fatty acid and retinol-binding protein gene *Ab-FAR-1* in the body wall of *A. besseyi* [50]. The gene *Dd-flp-1* is expressed in the circumpharyngeal nerve ring, retrovesicular ganglion, and ventral cord of *D. destructor* [51]. Some effectors are expressed differently at different development stages of plant-parasitic nematodes, which could be linked to their characteristics and biological functions. For instance, the localization of serine carboxypeptidase Rs-cps in the esophageal gland of *R. similis* may be related to destroying the host immune response and promoting the establishment of parasitic relationship, while the localization of Rs-cps in eggs and reproductive system may indicate that it may play an important role in the development and reproduction of nematodes [52].

### 2.2. Localization of Migratory Plant Endoparasitic Nematode Effectors in Plant Cells

The targeting efficacy of plant nematode effectors within plant cells underscores their function [53], emphasizing the crucial significance of understanding their localization within plant cells when investigating effector function. Subcellular localization and histological sectioning are the primary methods for observing the action sites of effector proteins within plant tissues [54]. Additionally, bioinformatics can be utilized to analyze and predict the localization of effector proteins in plants, although validation through corresponding experiments remains necessary. Numerous studies have demonstrated that effectors from MPENs primarily target the nucleus and cytoplasm of plant cells. The protein AbSCP1 acts on the nucleus of host plants, which may be related to its acceleration of protein degradation of host plants, thus promoting the feeding and parasitism of *A. besseyi* [44]. The protein BxSCD1 is localized within the nucleus and cytoplasm, with its cytoplasmic presence being essential for *B. xylophilus* to suppress programmed cell death induced by PsXEG1 [48]. PSORT II software predicted that Ab-FAR-1 was located in the plant nucleus with 82.6% possibility. Further subcellular localization experiments showed that fluorescence signals were found in the nucleus and cytoplasm of *Arabidopsis thaliana* and tobacco leaves, and its localization was closely related to the host’s defense response [50]. Furthermore, the effector Ppen10370 of *Pratylenchus penetrans* exhibited fluorescence signals on the endoplasmic reticulum and nuclear membrane of *Nicotiana benthamiana* [55].

## 3. The Functions of Effectors in Migratory Plant Endoparasitic Nematodes

The functions of plant-parasitic nematode effectors can be summarized into four major categories: (a) effectors involved in the degradation and modification of plant cell walls; (b) effectors influencing host immune responses; (c) effectors promoting nematode reproduction; (d) effectors inducing and maintaining nematode feeding sites by modulating host plant developmental pathways. Root-knot nematodes (RKN, *Meloidogyne* spp.) and cyst nematodes (CNs, *Globodera* and *Heterodera* spp.) have the ability to modulate the developmental pathways of host plants, thereby eliciting the formation of feeding sites conducive to their growth and development, such as giant cells and syncytium formation [56,57]. Distinguished from sedentary plant-parasitic nematodes like RKN and CNs, MPENs do not establish stationary feeding sites, and their effector functionalities predominantly encompass the top three categories. It remains uncertain whether there exist effectors analogous to those instigating and perpetuating nematode feeding sites through the modulation of host plant developmental pathways. Reny et al. compared certain effectors inferred from *R. similis* with those of other plant-parasitic nematodes, revealing an absence of effectors linked to the formation and development of feeding sites in the genome of *R. similis* [58].

### 3.1. Effectors Involved in the Degradation and Modification of Plant Cell Walls

The cell wall serves as the primary barrier in plants against pathogenic infections. Throughout the extensive co-evolutionary history between plants and pathogens, the latter have developed the capability of secreting cell wall degrading enzymes (CWDEs) for various polysaccharide components in plant cell walls, such as pectinase, cellulase, hemicellulase, lignin degrading enzymes, and so on [59]. CWDEs play a key role in the successful infection of plants by plant-parasitic nematodes, with their coding genes being prevalent across the genomes of such nematodes [60]. In 1998, the β-1, 4-glucan endonuclease gene of cellulase of animal origin was first found in the esophageal gland of the cyst nematodes [61]. At present, horizontal gene transfer (HGT) stands as the primary mechanism through which plant-parasitic nematodes acquire cell wall degrading enzymes [62]. *B. xylophilus* can be cultivated by fungi, and the resemblance between its cellulase and fungal cellulase is more pronounced, suggesting potential acquisition through HGT from fungal sources [45]. Nicol et al. disclosed that a gene encoding a plant cell wall degrading enzyme in *Pratylenchus* spp. might have been obtained by HGT [63]. The evolutionary analysis of Rs-eng-1 indicates its association with the cellulase secreted by the bacterial species *Bacillus subtilis* and *Erwinia carotovora*, thereby possibly indicating a bacterial origin [64]. With the profound investigation into nematode effectors, an increasing array of genes encoding cell wall degrading enzymes from MPENs have been reported. Lander et al. identified a novel cell wall degrading enzyme, β-glucuronidase, in the ESTs of *H. oryzae* [65]. The cellulase Pv-eng-1 from *P. vulnus* and two pectate lyases, Bx-pel-1 and Bx-pel-2, from *B. xylophilus* can degrade the cell walls of host plants, facilitating nematode invasion and migration [49,66]. Some effectors with the function of modifying plant cell walls also play an important role in the infection and parasitism of plant-parasitic nematodes. For example, expansin can destroy the covalent bond of plant cell walls, thus relaxing plant cell walls, which is more conducive to the infection of nematodes [67]. Expansin-like proteins have been reported on two MPENs, namely *B. xylophilus* (Bx-EXPB1, Bx-EXPB2, and Bx-EXPB3) [68] and *D. destructor* (DD-EXP-1 and DD-EXP-2) [69].

### 3.2. Effectors Influencing Host Immune Responses

Two-layer immune systems of plants, PAMP-triggered immunity response (PTI) and effector-triggered immunity (ETI), are the main means for plants to resist pathogen infection [70]. Thus far, our understanding of plant-parasitic nematode-associated molecular patterns and the signaling pathways triggered by PAMPs from plant-parasitic nematodes remains limited [71]. The role of MPENs in modulating plant immune responses has garnered increasing attention in recent studies. Indeed, the impact of plant-parasitic nematode effectors on Reactive Oxygen Species (ROS) levels, callose deposition, plant defense gene expression, and the formation of host necrotic spots serves as a significant indicator for assessing whether these effectors promote or inhibit the immune response of plants [72]. Many effectors of MPENs have been proved to effectively suppress the immune response of plants, including the venom allergen-like protein RsVAP [73] and the chorismate mutase RsCM [74] of *R. simili*; effectors BxSCD1 [48], BxSCD3 [75], BxSCD5 [76], BxICD1 [77], and BxLip-3 [78] of *B. xylophilus*; the fatty acid and vitamin A binding protein Ab-FAD-1 [50] of *A. besseyi*, and various genes encoding tissue proteases such as *Pc-CZ*, *Pc-CD*, *Pc-CB*, and *Pc-CL*, along with membrane-associated protein genes *PC-NEX-1* and *PC-NEX-2* from *P. coffeae* [79,80,81], among others. Research has revealed that the inhibitory effect of effector proteins on plant immune responses requires reaching a certain concentration threshold. Li et al. found that the effector protein Bx-FAR-1 from *B. xylophilus* could only suppress *Nicotiana benthamiana* necrosis induced by INF1 when its concentration reached 100 nmol/L [82]. Plant-parasitic nematodes not only have the ability to suppress the host’s immune responses but also induce immune responses in the host. Reported effectors capable of inducing host immune responses from migratory plant endoparasitic nematodes include the apoptotic protein BxCDP1 [83], the venom allergen-like protein Bx-vap-1 [84], and the sphingolipid activator protein BxSapB1 [85] from *B. xylophilus*, as well as the ATP synthase Ab-atps from *A. besseyi* [86].

### 3.3. Effectors Promoting Nematode Reproduction

In migratory parasitic nematodes, various effectors have been shown to be closely associated with nematode reproduction, primarily focusing on peptidases and calcium-binding proteins as the two principal effector categories. The serine carboxypeptidases Rs-scp-1 and AbSCP1 may potentially facilitate nematode degradation of host plant proteins and nutrient acquisition, thereby fostering their reproduction and parasitism [28,44,52]. Ab-CB-1 possesses proteolytic properties, enabling the degradation of carrot callus tissue to provide sustenance for its growth and reproduction, as evidenced in the proliferation of *A. besseyi* [87]. AbCRT affects the feeding and reproduction of *A. besseyi*, and downregulating the expression of this gene can reduce the frequency of nematode feeding, consequently decreasing their reproductive capacity [88]. Similar effectors include BxCRT [34], Bx-cpl [89], and Rs-CRT [90]. Furthermore, cytochrome P450 is also crucial for the reproduction of plant-parasitic nematodes, as evidenced by a significant reduction in the reproductive capacity of pine wood nematodes upon silencing the Bxcyp-33C9 gene [91].

The tissue localization of identified MPEN effectors involved in this paper is summarized in Table 1.

## 4. The Interaction between Effectors of Migratory Plant Endoparasitic Nematodes and Host Plants

In the theoretical investigation of the interaction mechanisms between plant pathogens and host plants, a plethora of models and hypotheses have emerged, including the gene-for-gene hypothesis, the pattern-triggered immunity hypothesis, the Zigzag model, and the decoy model [70,95,96,97]. Among these, the “zig-zag model” presently occupies a central position in research, with a multitude of studies attesting to its relevance in elucidating the interaction dynamics between plant-parasitic nematodes and host plants [98]. Pattern recognition receptors (PRRs) in plants recognize pathogen-associated molecular patterns (PAMPs) and activate the basic immune response PTI. Pathogens enhance their virulence by secreting effectors to interfere with PTI, which leads to effector-triggered susceptibility (ETS). At the same time, plants sense the effector effects of pathogens through nucleotide binding and leucine-rich repeat protein (NB-LRR protein) and start a specific immune response ETI. The interaction among PTI, ETS, and ETI is summarized as the zig-zag model [70]. The intricate interaction between the MPEN effectors and host plants is crucial in meticulously orchestrating the infection process and in the fine-tuning of the host’s physiological responses. Exploring the target proteins of plant-parasitic nematode effectors in the host will facilitate the elucidation of the pathogenic mechanisms of plant-parasitic nematodes and the formulation of more targeted control strategies [99,100,101]. Currently, yeast two-hybrid (Y2H) assays serve as the primary method for screening plant-parasitic nematode effectors interacting with receptor proteins within the host. To mitigate the risk of false positives in receptor protein identification, additional techniques such as bimolecular fluorescent complementation (BiFC) or co-immunoprecipitation (Co-IP) are often employed for validation [54]. Among plant-parasitic nematodes investigations into the interaction proteins of effectors in host plants predominantly focus on sedentary parasitic nematodes [102], while studies on migratory parasitic nematodes remain relatively sparse.

The cellular microfilament component of the cytoskeleton exerts a significant influence on the mediation of plant immune signaling pathways. In recent years, progress has been made in understanding plant cytoskeleton–pathogen interactions, and some effectors can regulate plant immune responses by influencing the cellular microfilament skeleton [103,104]. Ding et al. reported that *A. besseyi* can interact with the AtADF3 of *Arabidopsis thaliana* by secreting Ab-FAR-1, disrupting the actin-depolymerizing function of AtADF3, thereby inhibiting plant PTI. This study reveals, for the first time in plant-parasitic nematodes, a novel mechanism by which effectors suppress plant disease resistance by influencing the microfilament skeleton, providing fresh insights into the interaction between plant-parasitic nematode effectors and host plants [50]. The effector of plant-parasitic nematodes interacts with host plant defense-related proteins, subsequently influencing the expression of host plant defense-related genes, thereby modulating plant defense responses. Zhang et al. conducted a screening of the interaction protein PtCyP1 from *B. xylophilus* BxML1 in *Pinus thunbergii* through yeast two-hybrid and immunoprecipitation techniques. They elucidated that this interaction suppressed the plant defense response by modulating the expression of PtCyP1. Notably, PtCyP1 exhibited substantial expression levels during *B. xylophilus* infection in pine trees, whereas BxML1 repressed the expression of PtCyP1 [92]. Additionally, the effector Ab-atps, secreted by *A. besseyi*, interacts with the rice defense gene *OsRLK3*, eliciting the self-defense response of rice. Subsequently, the secretion of OsRLK3 is suppressed by Ab-atps in the later stages. Further investigation is required to ascertain whether Ab-atps inhibits or evades the defense response mediated by OsRLK3 [86]. Some effectors of plant-parasitic nematodes can regulate the metabolic pathway of plants and interact with host plants by simulating the host plant protein [105]. Wen et al. discovered for the first time that PtACO1, an ethylene-forming enzyme in pine trees, can interact with BxSCD1, an effector of *B. xylophilus*, and speculated that its interaction interfered with ethylene biosynthesis [48]. After secreting RsCM into the plant cytoplasm, *R. similis* may competitively inhibit the synthesis of salicylic acid (SA) in plant cells by vying for chorismic acid with chorismate mutase of the host plant, thereby suppressing plant defense responses [73]. HoCM and HoICM of *H. oryzae* may interfere with the synthesis of salicylic acid in the host plant, thus reducing the secondary metabolism level of the plant and inhibiting the plant immune response [106]. It is noteworthy that the ubiquitin pathway is integral to the interaction between plant-parasitic nematodes and host plants. There are some effectors in plant-parasitic nematodes that can hijack the ubiquitination system of the host or have ubiquitinated ligase activity in other ways, thus regulating the expression of defense-related factors of the host [107]. Paulo et al. identified a significant upregulation of ubiquitin-related genes and the distinct localization of the *Pratylenchus penetrans* effector Ppen10370 within the endoplasmic reticulum in plants expressing this effector. They postulated its potential involvement in the host’s ubiquitination pathway and its impact on the plant’s immune response, although the precise mechanism warrants further investigation [55]. Hu et al. demonstrated the interaction between BxCDP1 and *Pinus thunbergii* protein PtRHF1, potentially functioning as a plant E3 ligase and engaging in the ubiquitination process of BxCDP1 within plants, thus stimulating plant immunity [108].

The similar effector between different plant-parasitic nematodes may have high similarity in function, but it shows diversity in the mechanism of regulating host defense response. Li et al. identified the protein RsVAP of *R. similis*, which interacted with LeRabAld in the tomato, and postulated that their interaction may interfere with the role of LeRabAld in vesicle transport. Additionally, they also discovered that RsVAP from *R. similis* and sedentary plant-parasitic nematodes can suppress the plant’s defense response in function; however, these proteins had different interaction host targets [73]. As an elicitor, Bx-vap-1 induces the host’s defense response and plays a crucial role in the interaction between *B. xylophilus* and its host pine tree [84]. Both *B. xylophilus* and *R. similis* belong to migratory endoparasitic nematodes, yet their VAPs exhibit diametrically opposed functions in the host’s defense response. BxKU1 and BxKU2 are Kunitz-type protease inhibitors identified in *B. xylophilus*. The former has been demonstrated to interact with PtCel2 and TLP4 of *Pinus thunbergii*, potentially disrupting the SA pathway, while the latter interacts with an extensin-like protein associated with PR10 of *P. thunbergii*. Through the distinct mechanisms of action of these two effectors, *B. xylophilus* inhibits the defense response of host plants [93]. MPENs have evolved a variety of strategies to overcome plant immune response to establish parasitic relationship, which just indicates the complexity of the interaction between MPENs and host plants.

The effectors with host targets are listed in Table 2.

## 5. Outlook

In the realm of plant-parasitic nematodes, most research efforts have been focused on sedentary plant-parasitic nematodes such as root-knot nematodes and cyst nematodes, leaving the MPENS investigation relatively scant, although to some extent, they exert a greater impact on crop quality and yield than some sedentary plant-parasitic nematodes. Therefore, identifying targets for their control has become one of the key directions in research. In recent years, both domestic and international scholars have made headway through transcriptomic analyses of MPENS, unveiling numerous effector transcripts. However, compared to sedentary parasitic nematodes, comprehensive investigations remain sparse. Presently, only a handful of effectors have been elucidated in terms of functionality, with a considerably limited understanding of their roles during the invasion of host plants by MPENs. Thus, many effectors await identification and further scrutiny. Furthermore, research on MPEN effectors have largely focused on the exploration and functional analysis of homologous effectors, with minimal attention devoted to the discovery of specific effectors dissimilar to known ones. Moving forward, there is a pressing need to intensify efforts in studying MPEN effectors, striving to unearth additional candidate effectors and conducting pertinent identification and functional analyses. Effectors have long been a focal point in research on MPENs, particularly concerning the identification of their targets and interactions within plants. These findings serve as foundational knowledge for a deeper understanding of the molecular mechanisms underlying the interaction between MPENs and plants, thereby facilitating the elucidation of the pathogenic mechanisms of these nematodes. Currently, our exploration and investigation of interacting proteins of MPEN effectors remain insufficient. This area warrants significant advancement in future research efforts focused on MPENs.

## Figures and Tables

**Table 1:** The localization of migratory plant endoparasitic nematode effectors.

Nematode	Effector ID	Tissue Localization	References
*Radopholus similis*	Rs-Pel-1/2/3/4/5	esophageal glands	[43]
	Rs-scp-1	procorpus, esophageal glands, and intestine	[28]
	Rs-cps	esophageal glands, ovaries, testes, vas deferens, and egg	[52]
	Rs-eng-1	esophageal glands	[64]
	RsVAP	esophageal glands	[73]
	RsCM	esophageal glands	[74]
	Rs-CRT	esophageal glands, gonad, intestine, and egg	[90]
*Pratylenchus penetrans*	Pp-pme	esophageal glands	[47]
	Ppen10370	esophageal glands	[55]
*Pratylenchus vulnus*	Pv-eng-5	intestine	[49]
*Pratylenchus coffeae*	Pc-CZ	esophageal glands	[79]
	Pc-CD	esophageal glands	[79]
	Pc-CB	esophageal glands	[80]
	Pc-CL	esophageal glands	[80]
	Pc-NEX-1	esophageal glands and egg	[81]
	Pc-NEX-2	esophageal glands	[81]
*Ditylenchus destructor*	DdVAP2	subventral esophageal glands	[46]
	Dd-flp-1	circumpharyngeal nerve ring, retrovesicular ganglion and ventral cord	[51]
	DD-EXP-1, DD-EXP-2	esophageal glands	[69]
*Bursaphelenchus xylophilus*	Bx-eng-1	esophageal glands	[45]
	BxSCD1	dorsal esophageal gland and intestine	[48]
	Bx-pel-1, Bx-pel-2	esophageal glands	[66]
	BxICD1	esophageal glands	[77]
	BxLip-3	esophageal glands and intestine	[78]
	BxCDP1	dorsal esophageal gland and intestine	[83]
	BxSapB1	subventral esophageal glands	[85]
	Bx-cpl	intestine, egg, and seminal vesicle	[89]
	BxML1	dorsal esophageal gland and intestine	[92]
	Bx-vap-1	esophageal glands	[84]
	BxKU1	esophageal glands	[93]
	BxKU2	esophageal glands and ovaries	[93]
	Bx-FAR-1	glandular tissue, intestine, and seminal vesicles	[94]
*Aphelenchoides besseyi*	AbSCP1	esophageal glands	[44]
	Ab-FAR-1	body wall	[50]
	Ab-atps	esophageal glands and reproductive system	[86]
	Ab-CB-1	intestine	[87]
	AbCRT	esophageal glands and gonad	[88]
*Hirschmanniella Oryzae*	*β*-mannanase	esophageal glands	[65]
	HoCM	esophageal glands	[65]

**Table 2:** Host targets of effectors in migratory plant endoparasitic nematodes.

Nematode	Effector	Effector Subtype	Interactive Host	Interactive Protein	References
*Radopholius similis*	RsVAP	venom allergen-like protein	*Lycopersicon esculentum*	LeRabA1d	[73]
*Bursaphelenchus xylophilus*	BxSCD1	-	*Pinus thunbergii*	PtACO1	[48]
*Bursaphelenchus xylophilus*	BxLip-3	lipase	*Pinus thunbergii*	PtChia1-3, PtChia1-4	[78]
*Bursaphelenchus xylophilus*	BxML1	ML proteins	*Pinus thunbergii*	PtCyP1	[92]
*Bursaphelenchus xylophilus*	BxCDP1	-	*Pinus thunbergii*	PtRHF1	[108]
*Bursaphelenchus xylophilus*	BxKU1	Kunitz-type protease inhibitors	*Pinus thunbergii*	TLP4, PtcysP, PtCel2, phospho-2-dehydro-3-deoxyheptonate aldolase 1	[93]
*Bursaphelenchus xylophilus*	BxKU2	Kunitz-type protease inhibitors	*Pinus thunbergii*	TLP4, extensin-like protein, PtChia1-1, PtMYB6	[93]
*Aphelenchoides besseyi*	Ab-FAR-1	Fatty acid and retinol binding proteins	*Arabidopsis thaliana*	AtADF3	[50]
*Aphelenchoides besseyi*	Ab-atps	ATP synthase gene	*Oryza sativa*	OsRLK3	[86]
*Pratylenchus coffeae*	PC-NEX-1	annexin gene	*Zea mays*	Zm Pi Fi	[81]

## Data Availability

Not applicable.

## References

[1] Elling A.A. (2013). Major emerging problems with minor *Meloidogyne* species. Phytopathology.

[2] Mathew R., Opperman C.H. (2020). Current insights into migratory endoparasitism: Deciphering the biology, parasitism mechanisms, and management strategies of key migratory endoparasitic phytonematodes. Plants.

[3] Perrine-Walker F. (2019). Interactions of endoparasitic and ectoparasitic nematodes within the plant root system. Funct. Plant Biol..

[4] Jones J.T., Haegeman A., Danchin E.G., Gaur H.S., Helder J., Jones M.G., Kikuchi T., Manzanilla-López R., Palomares-Rius J.E., Wesemael W.M. (2013). Top 10 plant-parasitic nematodes in molecular plant pathology. Mol. Plant Pathol..

[5] Yang S.-H., Zhao L.-R., Ding S., Tang S.-Q., Chen C., Zhang H.-X., Xie H. (2022). Study on burrowing nematode, *Radopholus similis*, pathogenicity test system in tobacco as host. J. Integr. Agric..

[6] Xie H. (2006). *Radopholus similis* and its detection and epidemic prevention control. Plant Quar..

[7] Singh R., Phulera S., Singh R.D. (2015). Plant parasitic nematodes: The hidden enemies of farmers. Environmental Issues for Socio-Ecological Development.

[8] Du Y., Zhou J., Liu B.-Y., Lei Q.-W. (2020). A checklist of species of the genus *Pratylenchus* and its Chinese species. Plant Quar..

[9] Gough E.C., Owen K.J., Zwart R.S., Thompson J.P. (2020). A systematic review of the effects of arbuscular mycorrhizal fungi on root-lesion nematodes, *Pratylenchus* spp.. Front. Plant Sci..

[10] Back M. (2009). *Pratylenchus* (Nematoda: Pratylenchidae): Diagnosis, biology, pathogenicity and management. Nematology Monographs and Perspectives Volume 6. Plant Pathol..

[11] Ye S., Zeng R., Zhou J., An M., Ding Z. (2020). Molecular characterization of *Ditylenchus destructor* voltage-gated calcium channel α1 subunits and analysis of the effect of their knockdown on nematode activity. Biochimie.

[12] Song W., Dai M., Shi Q., Liang C., Duan F., Zhao H. (2023). Diagnosis and characterization of *Ditylenchus destructor* isolated from Mazus japonicus in China. Life.

[13] Ren B.-Z., Tang L.-H. (2020). Progress in quarantine pests of *Bursaphelenchus xylophilus*. J. Jilin Agric. Univ..

[14] Vicente C., Espada M., Vieira P., Mota M. (2012). Pine wilt disease: A threat to European forestry. Eur. J. Plant Pathol..

[15] Xie J.-L., Yang F., Wang Y.-P., Peng Y.-L., Ji H.-L. (2019). Studies on the efficiency of different inoculation methods of rice white-tip nematode, *Aphelenchoides besseyi*. Nematology.

[16] Kanzaki N., Giblin-Davis R.M., Scheffrahn R.H., Taki H., Esquivel A., Davies K.A., Herre E.A. (2012). Reverse taxonomy for elucidating diversity of insect-associated nematodes: A case study with termites. PLoS ONE.

[17] Meyer M.C., Favoreto L., Klepker D., Marcelino-Guimarães F.C. (2017). Soybean green stem and foliar retention syndrome caused by *Aphelenchoides besseyi*. Trop. Plant Pathol..

[18] Favoreto L., Meyer M.C., Calandrelli A., Maia da Silva M.C., Aleandro da Silva S., Machado A.C.Z. (2020). *Aphelenchoides besseyi* parasitizing common bean in Brazil. Plant Dis..

[19] He J., Liu S.-T., Chen C., Ding S.-W., Xie H., Xu C.-L. (2021). A loop-mediated isothermal amplification method for detection of *Hirschmanniella oryzae* based on rDNA-ITS sequences. Acta Phytopathol. Sin..

[20] Karakaş M. (2007). Life cycle and mating behavior of *Helicotylenchus multicinctus* (Nematoda: Hoplolaimidae) on excised Musa cavendishii roots. Biologia.

[21] Abd-Elgawad M.M.M., Ansari R., Rizvi R., Mahmood I. (2020). Plant-parasitic nematodes andtheir biocontrol agents: Current status and future vistas. Management of Phytonematodes: Recent Advances and Future Challenges.

[22] Pires D., Vicente C.S.L., Menéndez E., Faria J.M.S., Rusinque L., Camacho M.J., Inácio M.L. (2022). The fight against plant-parasitic nematodes: Current status of bacterial and fungal biocontrol agents. Pathoges.

[23] Gong L.-E., Ying S.-M., Zhang Y.-F., Wang J.-Y., Sun G.-C. (2023). Strategies for exogenous RNA delivery in RNAi-mediated pest management. Chin. J. Biotechnol..

[24] Feng J.-Y., Li C.-K., Ding S.-L., Liu J., Yin X.-M., An S.-H., Na R.-S., Liu X.-G. (2022). Research advances on the application of RNA interference in agricultural disease and pest control. Chin. J. Pestic. Sci..

[25] Gautam P., Kumar R., Feroz Z., Vijayaraghavalu S., Kumar M., Singh R.L., Mondal S., Parihar A., Singh P.K. (2022). RNA interference technology in plants: Mechanisms and applications in cropimprovement. Plant Genomics for Sustainable Agriculture.

[26] Chen S.-Y. (2020). The Use of RNA Interference in Enhancing Plant Resistance against Nematodes. J. Bot. Res..

[27] Mwaka H.S., Bauters L., Namaganda J., Marcou S., Bwesigye P.N., Kubiriba J., Smagghe G., Tushemereirwe W.K., Gheysen G. (2023). Transgenic East African Highland Banana Plants Are Protected against *Radopholus similis* through Host-Delivered RNAi. Int. J. Mol. Sci..

[28] Huang X., Xu C.-L., Chen W.-Z., Chen C., Xie H. (2017). Cloning and characterization of the first serine carboxypeptidase from a plant parasitic nematode, *Radopholus similis*. Sci. Rep..

[29] Ding S.-W., Wang D.-W., Xu C.-L., Yang S.-H., Cheng X., Peng X.-F., Chen C., Xie H. (2021). A new fungus-mediated RNAi method established and used to study the fatty acid and retinol binding protein function of the plant-parasitic nematode *Aphelenchoides besseyi*. RNA Biol..

[30] Wang D.-W., Xu C.-L., Ding S.-W., Huang X., Cheng X., Zhang C., Chen C., Xie H. (2018). Identification and function of FAR protein family genes from a transcriptome analysis of *Aphelenchoides besseyi*. Bioinformatics.

[31] Wang C., Guo K. (2024). Application of dsRNA in the Pine Wood Nematode, *Bursaphelenchus xylophilus*. Methods Mol. Biol..

[32] Liu X., Zhou X., Zhou L., Hu J., Guo K. (2022). Application of RNA Interference in the Pinewood Nematode, *Bursaphelenchus xylophilus*. J. Vis. Exp..

[33] Kang J.S., Lee D.W., Koh Y.H., Lee S.H. (2011). A soluble acetylcholinesterase provides chemical defense against xenobiotics in the pinewood nematode. PLoS ONE.

[34] Li X.-D., Zhuo K., Luo M., Sun L.-H., Liao J.-L. (2011). Molecular cloning and characterization of a calreticulin cDNA from the pinewood nematode *Bursaphelenchus xylophilus*. Exp. Parasitol..

[35] Hogenhout A.S., Van der Hoorn L.A.R., Terauchi R., Kamoun S. (2009). Emerging concepts in effector biology of plant-associated organisms. Mol. Plant Microbe Interact..

[36] Davis E.L., Hussey R.S., Mitchum M.G., Baum T.J. (2008). Parasitism proteins in nematode–plant interactions. Curr. Opin. Plant Biol..

[37] Vieira P., Myers R.Y., Pellegrin C., Wram C., Hesse C., Maier T.R., Shao J., Koutsovoulos G.D., Zasada I., Matsumoto T. (2021). Targeted transcriptomics reveals signatures of large-scale independent origins and concerted regulation of effector genes in *Radopholus similis*. PLoS Pathog..

[38] Hu L.-L., Lin B.-R., Wang H.-H., Chen J.-S., Liao J.-L., Zhuo K. (2023). Transcriptome and Candidate Effectors Analysis of *Pratylenchus brachyurus*. Biol. Bull..

[39] Huang X., Xu C.-L., Yang S.-H., Li J.-Y., Wang H.-L., Zhang Z.-X., Chen C., Xie H. (2019). Life-stage specific transcriptomes of a migratory endoparasitic plant nematode, *Radopholus similis* elucidate a different parasitic and life strategy of plant parasitic nematodes. Sci. Rep..

[40] Channale S., Kalavikatte D., Thompson J.P., Kudapa H., Bajaj P., Varshney R.K., Zwart R.S., Thudi M. (2021). Transcriptome analysis reveals key genes associated with root-lesion nematode *Pratylenchus thornei* resistance in chickpea. Sci.Rep..

[41] Wang F., Li D.-L., Wang Z.-Y., Dong A.-R., Liu L.-H., Wang B.-Y., Chen Q.-L., Liu X.-H. (2017). Transcriptomic analysis of the rice white tip nematode, *Aphelenchoides besseyi* (Nematoda: Aphelenchoididae). PLoS ONE.

[42] Rosso M.N., Hussey R.S., Davis E.L., Smant G., Baum T.J., Abad P., Mitchum M.G., Martin F., Kamoun S. (2011). Nematode effector proteins: Targets and functions in plant parasitism. Effectors in Plant–Microbe Interactions.

[43] Huang X. (2018). Life Stage-Specific Transcriptomes Analysis of *Radopholus similis* and RNAi Analysis of Six Pathogenic Genes. Ph.D. Thesis.

[44] Huang X., Chi Y.-K., Birhan A.A., Zhao W.-B., Qi R.-D., Peng D.-L. (2022). The new effector AbSCP1 of foliar nematode (*Aphelenchoides besseyi*) is required for parasitism rice. J. Integr. Agric..

[45] Kikuchi T., Jones J.T., Aikawa T., Kosaka H., Ogura N. (2004). A family of glycosyl hydrolase family 45 cellulases from the pine wood nematode *Bursaphelenchus xylophilus*. FEBS Lett..

[46] Chang Q., Yang Y.-W., Hong B., Zhao M.-X., Han S.-S., Zhang F., Peng H., Peng D.-L., Li Y.-M. (2023). A variant of the venom allergen-like protein, DdVAP2, is required for the migratory endoparasitic plant nematode *Ditylenchus destructor* parasitism of plants. Front. Plant Sci..

[47] Vicente C.S.L., Nemchinov L.G., Mota M., Eisenback J.D., Kamo K., Vieira P. (2019). Identification and characterization of the first pectin methylesterase gene discovered in the root lesion nematode *Pratylenchus penetrans*. PLoS ONE.

[48] Wen T.-Y., Wu X.-Q., Hu L.-J., Qiu Y.-J., Rui L., Zhang Y., Ding X.L., Ye J.R. (2021). A novel pine wood nematode effector, BxSCD1, suppresses plant immunity and interacts with an ethylene-forming enzyme in pine. Mol. Plant Pathol..

[49] Fanelli E., Troccoli A., Picardi E., Pousis C., Luca D.F. (2014). Molecular characterization and functional analysis of four beta-1,4-endoglucanases from the root-lesion nematode *Pratylenchus vulnus*. Plant Pathol..

[50] Ding S.-W., Cheng X., Wang D.-W., Chen C., Yang S.-H., Wang J.-F., Xu C.-L., Xie H. (2022). *Aphelenchoides besseyi* Ab-FAR-1 Interacts with Arabidopsis thaliana AtADF3 to interfere with actin cytoskeleton, and promotes nematode parasitism and pathogenicity. Int. J. Mol. Sci..

[51] Peng H., Hu W.-Q., Huang W.-K., He W.-T., Peng D.-L. (2011). Cloning and localization analysis of a novel FMRFamide-like neuropeptide Gene (Dd-flp-1) from Migration Plant-parasitic Nematode (*Ditylenchus destructor*) on Sweetpotato in China. J. Agric. Biotechnol..

[52] Wang K., Li Y., Huang X., Wang D.-W., Xu C.-L., Xie H. (2016). The cathepsin S cysteine proteinase of the burrowing nematode *Radopholus similis* is essential for the reproduction and invasion. Cell Biosci..

[53] Jaouannet M., Rosso M.N. (2013). Effectors of root sedentary nematodes target diverse plant cell compartments to manipulate plant functions and promote infection. Plant Signal Behav..

[54] Hu L.-L., Zhuo K., Lin B.-R., Liao J.-L. (2016). The research progress of methods on function analysis of effectors from plant-parasitic nematode. Chin. Biotechnol..

[55] Vieira P., Vicente C.S.L., Branco J., Buchan G., Mota M., Nemchinov L.G. (2021). The root lesion nematode effector Ppen10370 is essential for parasitism of *Pratylenchus penetrans*. Mol. Plant Microbe Interact..

[56] Vieira P., Gleason C. (2019). Plant-parasitic nematode effectors—Insights into their diversity and new tools for their identification. Curr. Opin. Plant Biol..

[57] Siddique S., Grundler F.M. (2018). Parasitic nematodes manipulate plant development to establish feeding sites. Curr. Opin. Microbiol..

[58] Reny M., Opperman C.H. (2019). The genome of the migratory nematode, *Radopholus similis*, reveals signatures of close association to the sedentary cyst nematodes. PLoS ONE.

[59] Wieczorek K. (2015). Cell Wall Alterations in Nematode-Infected Roots. Adv. Bot. Res..

[60] Bird D.M., Williamson V.M., Abad P., McCarter J., Danchin E.G., Castagnone-Sereno P., Opperman C.H. (2009). The genomes of root-knot nematodes. Annu. Rev. Phytopathol..

[61] Smant G., Stokkermans G.W.P.J., Yan Y.-T., de Boer J.-M., Baum T.J., Wang X.-H., Hussey S.R., Gommers J.F., Henrissat B., Davis L.E. (1998). Endogenous cellulases in animals: Isolation of β-1,4-endoglucanase genes from two species of plant-parasitic cyst nematodes. Proc. Natl. Acad. Sci. USA.

[62] Mitreva M., Smant G., Helder J. (2009). Role of horizontal gene transfer in the evolution of plant parasitism among nematodes. Methods Mol. Biol..

[63] Nicol P., Gill R., Fosu-Nyarko J., Jones M.G. (2012). de novo analysis and functional classification of the transcriptome of the root lesion nematode, *Pratylenchus thornei*, after 454 GS FLX sequencing. Int. J. Parasitol..

[64] Luo X., Chen G.-H., Dai L.-Y., Lu C.-H., Yang Y.-H., Xie B.-Y. (2008). Cloning and characterization of β-1,4-endoglucanse gene from burrowing nematode *Radopholus simili*. Acta Hortic. Sin..

[65] Lander B., Haegeman A., Kyndt T., Gheysen G. (2014). Analysis of the transcriptome of *Hirschmanniella oryzae* to explore potential survival strategies and host-nematode interactions. Mol. Plant Pathol..

[66] Kikuchi T., Shibuya H., Aikawa T., Jones J.T. (2006). Cloning and characterization of pectate lyases expressed in the esophageal gland of the pine wood nematode *Bursaphelenchus xylophilus*. Mol. Plant-Microbe Interact..

[67] Cosgrove J.D. (2000). Loosening of plant cell walls by expansins. Nature.

[68] Lee D.W., Seo J.B., Kang J.S., Koh S.H., Lee S.H., Koh Y.H. (2012). Identification and characterization of expansins from *Bursaphelenchus xylophilus* (Nematoda: Aphelenchoididae). Plant Pathol. J..

[69] Peng H., Gao B.-L., Kong L.-A., Yu Q., Huang W.-K., He X.F., Long H.-B., Peng D.-L. (2017). Exploring the host parasitism of the migratory plant-parasitic nematode *Ditylenchus destructor* by expressed sequence tags analysis. PLoS ONE.

[70] Jones J.D.G., Dangl J.L. (2006). The plant immune system. Nature.

[71] Mantelin S., Thorpe P., Jones J.T. (2015). Suppression of plant defences by plant-parasitic nematodes. Adv. Bot. Res..

[72] Dodds P.N., Rathjen J.P. (2010). Plant immunity: Towards an integrated view of plant-pathogen interactions. Nat. Rev. Genet..

[73] Li J.-Y., Xu C.-L., Yang S.-H., Chen C., Tang S.-Q., Wang J.-F., Xie H. (2021). A venom allergen-like protein, RsVAP, the first discovered effector protein of *Radopholus similis* that inhibits plant defense and facilitates parasitism. Int. J. Mol. Sci..

[74] Yang S.-H., Li J.-Y., Yang S., Tang S.-Q., Wang H.-Z., Xu C.-L., Xie H. (2024). A chorismate mutase from *Radopholus similis* plays an essential role in pathogenicity. J. Integr. Agric..

[75] Hu L.-J., Wu X.-Q., Wen T.-Y., Qiu Y.-J., Rui L., Zhang Y., Ye J.-R. (2022). A *Bursaphelenchus xylophilus* Effector, BxSCD3, Suppresses Plant defense and contributes to virulence. Int. J. Mol. Sci..

[76] Hu L.-J., Wu X.-Q., Ding X.-L., Ye J.-R. (2021). Comparative transcriptomic analysis of candidate effectors to explore the infection and survival strategy of *Bursaphelenchus xylophilus* during different interaction stages with pine trees. BMC Plant Biol..

[77] Li Z.-W., Wang H.-H., Cao Y.-Q., Shan X.-L., He X.-X., Huang Q.-L., Zhuo K., Liao J.-L., Lin B.-R. (2024). A *Bursaphelenchus xylophilus* effector BxICD1 inducing plant cell death, concurrently contributes to nematode virulence and migration. Front. Plant Sci..

[78] Qiu Y.-J., Wu X.-Q., Wen T.-Y., Hu L.-J., Rui L., Zhang Y., Ye J.-R. (2023). The *Bursaphelenchus xylophilus* candidate effector BxLip-3 targets the class I chitinases to suppress immunity in pine. Mol. Plant Pathol..

[79] Xia Y.-H. (2022). Isolation and Identification of *Pratylenchus* Species and Function Study of Effector Proteins PC-CZ, PC-CD. Master’s Thesis.

[80] Hu Y.-J. (2022). Identification of *Pratylenchus* from Rhizosphere of Corn and Function Study of Two Genes of *P. coffeae*. Master’s Thesis.

[81] Sun M.R. (2023). Function Study of Effector Proteins PC-NEXs in *Pratylenchus coffeae*. Master’s Thesis.

[82] Li Y., Wu X.-Q., Hu L.-J. (2020). Prokaryotic expression and activity research of *Bursaphelenchus xylophilus* effector protein Bx-FAR-1. J. South Agric..

[83] Hu L.-J., Wu X.-Q., Li H.-Y., Wang Y.-C., Huang X., Wang Y., Li Y. (2020). BxCDP1 from the pine wood nematode *Bursaphelenchus xylophilus* is recognized as a novel molecular pattern. Mol. Plant Pathol..

[84] Li Y.-X., Wang Y., Liu Z.-Y., Jia X.-Z., Zhang X.-Y., Wang X., Lu Q. (2016). Functional analysis of the venom allergen-like protein gene from pine wood nematode *Bursaphelenchus xylophilus* using a baculovirus expression system. Physiol. Mol. Plant Pathol..

[85] Hu L.-J., Wu X.-Q., Li H.-Y., Zhao Q., Wang Y.-C., Ye J.-R. (2018). An effector, BxSapB1, induces cell death and contributes to virulence in the pine wood nematode *Bursaphelenchus xylophilus*. Mol. Plant-Microbe Interact..

[86] Wang H.-L., Xu C.-L., Chen C., Ding S.-W., Li J.-Y., Yang S.-H., Xie H. (2021). A novel ATPase gene, Ab-atps, plays an important role in the interaction of rice and white tip nematode, *Aphelenchoides besseyi*. Sci. Rep..

[87] Wang H.-L., Cheng X., Ding S.-W., Wang D.-W., Chen C., Xu C.-L., Xie H. (2018). Molecular identification and functional characterization of the cathepsin B gene (Ab-cb-1) in the plant parasitic nematode *Aphelenchoides besseyi*. PLoS ONE.

[88] Feng H., Wei L.-H., Chen H.-G., Zhou Y.-J. (2015). Calreticulin is required for responding to stress, foraging, and fertility in the white-tip nematode, *Aphelenchoides besseyi*. Exp. Parasitol.

[89] Xue Q., Wu X.-Q., Zhang W.-J., Deng L.-N., Wu M.-M. (2019). Cathepsin L-like cysteine proteinase genes are associated with the development and pathogenicity of pine wood nematode, *Bursaphelenchus xylophilus*. Int. J. Mol. Sci..

[90] Yu L., Wang K., Xie H., Wang Y.-T., Wang D.-W., Xu C.-L., Huang X., Wang D.-S. (2015). A nematode calreticulin, Rs-CRT, is a key effector in reproduction and pathogenicity of *Radopholus similis*. PLoS ONE.

[91] Qiu X., Yang L., Ye J., Wang W., Zhao T., Hu H., Zhou G. (2019). Silencing of cyp-33C9 Gene affects the reproduction and pathogenicity of the pine wood nematode, *Bursaphelenchus xylophilus*. Int. J. Mol. Sci..

[92] Zhang Y., Wen T.-Y., Wu X.-Q., Hu L.-J., Qiu Y.-J., Rui L. (2022). The *Bursaphelenchus xylophilus* effector BxML1 targets the cyclophilin protein (CyP) to promote parasitism and virulence in pine. BMC Plant Biol..

[93] Wen T.-Y., Wu X.-Q., Ye J.-R., Qiu Y.-J., Rui L., Zhang Y. (2023). Two novel *Bursaphelenchus xylophilus* kunitz effector proteins using different infection and survival strategies to suppress immunity in pine. Phytopathology.

[94] Li Y., Hu L.-J., Wu X.-Q., Ye J.-R. (2020). A *Bursaphelenchus xylophilus* effector, Bx-FAR-1, suppresses plant defense and affects nematode infection of pine trees. Eur. J. Plant Pathol..

[95] Flor H.H. (1971). Current status of the gene-for-gene concept. Annu. Rev. Phytopathol..

[96] Dangl J.L., Jones J.D. (2001). Plant pathogens and integrated defence responses to infection. Nature.

[97] van der Hoorn R.A., Kamoun S. (2008). From guard to decoy: A new model for perception of plant pathogen effectors. Plant Cell.

[98] Bauters L., Gheysen G. (2015). How the Plant-Parasitic Nematode Hirschmanniella oryzae Is Able to Subdue the Defense System of Rice: A Molecular Analysis.

[99] Abd-Elgawad M.M.M. (2022). Understanding molecular plant–nematode interactions to develop alternative approaches for nematode control. Plants.

[100] Abd-Elgawad M.M.M. (2022). Exploiting plant–phytonematode interactions to upgrade safe and effective nematode control. Life.

[101] Eves-van den Akker S., Stojilković B., Gheysen G. (2021). Recent applications of biotechnological approaches to elucidate the biology of plant–nematode interactions. Curr. Opin. Biotechnol..

[102] Bali S., Gleason C. (2024). Unveiling the diversity: Plant parasitic nematode effectors and their plant interaction partners. Mol. Plant Microbe Interact..

[103] Bhandari D.D., Brandizzi F. (2020). Plant endomembranes and cytoskeleton: Moving targets in immunity. Curr. Opin. Plant Biol..

[104] Kang Y.-S., Jelenska J., Cecchini N.M., Li Y.-J., Lee M.W., Kovar D.R., Greenberg J.T. (2014). HopW1 from *Pseudomonas syringae* disrupts the actin cytoskeleton to promote virulence in Arabidopsis. PLoS Pathog..

[105] Gheysen G., Mitchum M.G. (2011). How nematodes manipulate plant development pathways for infection. Curr. Opin. Plant Biol..

[106] Bauters L., Kyndt T., De Meyer T., Morreel K., Boerjan W., Lefevere H., Gheysen G. (2020). Chorismate mutase and isochorismatase, two potential effectors of the migratory nematode *Hirschmanniella oryzae*, increase host susceptibility by manipulating secondary metabolite content of rice. Mol. Plant Pathol..

[107] Kud J., Wang W., Gross R., Fan Y., Huang L., Yuan Y., Gray A., Duarte A., Kuhl J.C., Caplan A. (2019). The potato cyst nematode effector RHA1B is a ubiquitin ligase and uses two distinct mechanisms to suppress plant immune signaling. PLoS Pathog..

[108] Hu L.-J., Wu X.-Q., Wen T.-Y., Ye J.-R., Qiu Y.-J., Rui L., Zhang Y. (2022). The key molecular pattern BxCDP1 of *Bursaphelenchus xylophilus* induces plant immunity and enhances plant defense response via two small peptide regions. Front. Plant Sci..

